# The *Discodermia calyx *Toxin Calyculin A Enhances Cyclin D1 Phosphorylation and Degradation, and Arrests Cell Cycle Progression in Human Breast Cancer Cells

**DOI:** 10.3390/toxins3010105

**Published:** 2011-01-24

**Authors:** Jessica R. Edelson, David L. Brautigan

**Affiliations:** Center for Cell Signaling and Department of Microbiology, University of Virginia School of Medicine, Charlottesville, VA 22908, USA; Email: jrh9x@virginia.edu

**Keywords:** toxins, phosphatases, cyclin D1, calyculin A, okadaic acid, cantharidin

## Abstract

Cyclin D1 is a key regulator of the cell cycle that is over expressed in more than half of breast cancer patients. The levels of cyclin D1 are controlled primarily through post-translational mechanisms and phosphorylation of cyclin D1 at T286 induces its proteasomal degradation. To date, no studies have explored the involvement of phosphatases in this process. Here we treated human breast cancer cells with the structurally distinct toxins calyculin A, okadaic acid, and cantharidin, which are known to inhibit Ser/Thr phosphatases of the PPP family. At low nanomolar concentrations calyculin A induced T286 phosphorylation and degradation of cyclin D1 via the proteosome in MDA-MB-468 and MDA-MB-231 cells. Cyclin D1 degradation also was dose-dependently induced by okadaic acid and catharidin, implicating a negative regulatory role for type-2A phosphatases. These effects occurred without increasing phosphorylation of p70S6K, cyclin D3, or myosin light chain that were used as endogenous reporters of cellular PP2A and PP1 activity. A reverse phase phosphoprotein array analysis revealed increased phosphorylation of only 6 out of 33 Ser/Thr phosphosites, indicating selective inhibition of phosphatases by calyculin A. Calyculin A treatment induced cell cycle arrest in MDA-MB-468 and MCF-7 breast cancer cells. These findings suggest that a specific pool of type-2A phosphatase is inhibited by calyculin A leading to the degradation of cyclin D1 in human breast cancer cells. The results highlight the utility of toxins as pharmacological probes and points to the T286 cyclin D1 phosphatase inhibited by calyculin A as a possible target for chemotherapy to treat triple negative breast cancer.

## 1. Introduction

Cyclin D1 regulates the G_1_ to S progression of the cell cycle and is over expressed at the protein level in several cancers including ~50% of breast cancers, as seen by immunohistochemistry [1]. In the G_1_ phase of the cell cycle mitogens induce cyclin D1 transcription resulting in an increase in levels of cytoplasmic cyclin D1 protein [2]. The cyclin D1 protein binds to cyclin dependent kinase 4/6 (CDK4/6) forming a kinase complex that translocates into the nucleus. The cyclin D1/CDK4 complex, once activated, phosphorylates the tumor suppressor protein Rb, releasing it from the transcription factor E2F [3,4]. E2F facilitates the transcription of genes necessary for progression through the S phase of the cell cycle, including cyclin E and cyclin A. Following a peak during the G_1_ phase, levels of cyclin D1 drop during the S phase of the cell cycle. This decrease has been attributed to cyclin D1 phosphorylation at Thr286, causing export from the nucleus via CRM-1 [5]. Phosphorylation at Thr286 also promotes binding of cyclin D1 by the SCF complex and ubiquitination leading to degradation by the proteosome [6,7]. GSK3β has been implicated as the major kinase in phosphorylation of cyclin D1 at Thr286 [8]. However, GSK3β is active throughout the cell cycle and therefore cannot account for the cell cycle dependent change in cyclin D1 Thr286 phosphorylation. Furthermore, phosphorylation at Thr286 and proteosome degradation cannot fully account for cyclin D1 turnover. Wild type cyclin D1 has a half-life of 30 minutes, whereas a T286A mutant and a splice variant of cyclin D1 lacking the C-terminal tail and the Thr286 site (cyclin D1b), still are degraded but with slightly longer half-lives [9,10]. Control of the cyclin D1 degradation pathway is still incompletely understood. 

There is evidence that defects in cyclin D1 degradation might account for the cyclin D1 protein accumulation found in cancers. An *in vitro* screen of 76 deubiquitinating enzymes showed that one particular enzyme, USP2, reacted with monoubiquitinated cyclin D1. Over expression of USP2 in 293 cells stabilized cyclin D1 protein levels by preventing ubiquitin-mediated proteasomal degradation. Knockdown of USP2 in HCT116 colorectal cancer, MCF-7 breast cancer, and PC-3 prostate cancer cells destabilized cyclin D1 and reduced cell proliferation [11]. These results offer hope that approaches to control cyclin D1 protein levels by interfering with the degradation pathway might be effective in arresting tumor growth. 

Phosphorylation of cyclin D1 induces its proteasomal degradation, therefore protein phosphatases have a putative role in dephosphorylation cyclin D1 to prevent its degradation. To date there is little information about the identity of the protein phosphatase responsible for dephosphorylation of cyclin D1. In yeast, levels of the G_1_ cyclins (CLN1, CLN2, and HCS26) that correspond to mammalian cyclin D1 are regulated in part by the Ser/Thr phosphatase Sit4 [12]. In PC-3 human prostate cancer cells expression of a fusion protein of GFP with the non-catalytic N-terminus of the phosphatase PP6 (the human ortholog of Sit4) caused a G_1_ cell cycle arrest with a corresponding reduction in cyclin D1 levels [13]. This response was not mimicked by expression of a PP2A N-terminal/GFP fusion protein. These results suggested a specific role for PP6 in regulation of cyclin D1 levels, whether by direct dephosphorylation or another mechanism is unknown. 

Several toxins in nature have been found to potently inhibit the PPP family of Ser/Thr phosphatases that includes the type-2A phosphatases (PP2A, PP4 and PP6) and the type-1 phosphatase, PP1. These toxins include the polyketals okadaic acid and calyculin A, purified from marine sponges, cyclic peptides of the microcystin and nodularin groups produced by blue green algae, and the compound cantharidin, an epoxycyclohexane dicarboxylic anhydride produced by blister beetles. The toxins calyculin A, okadaic acid, and cantharidin were chosen for this study based on their cell permeability and chemical stability. These three toxins are reported to show preferential inhibition of type-2A phosphatases compared to type-1 phosphatases [14,15]. The aim of this study was to examine whether inhibition of type-2A phosphatases would promote degradation of cyclin D1 levels in human breast cancer cells, exposing a mechanism by which small molecule inhibitors may aid in suppression of tumor cell proliferation. We found that treating MDA-MB-468, MDA-MB-231, and MCF-7 breast cancer cells with these phosphatase inhibitory toxins decreased levels of cyclin D1. Unexpectedly, calyculin A induced proteosome degradation of cyclin D1 in MDA-MB-468 cells at more than an order of magnitude lower doses than the other toxins. These very low doses of calyculin A (<10 nM) increased Ser/Thr phosphorylation of only a few recognized endogenous substrates of PPP phosphatases, suggesting differential inhibition of subcellular pools of type 2A phosphatases.

## 2. Materials and Methods

### 2.1. Tissue Culture

MDA-MB-468 and MDA-MB-231 cells were maintained in L_15 _media (Gibco 11415) containing 10% fetal bovine serum at 37° in non-CO_2_ conditions. MCF-7 cells were maintained in MEM media (Gibco 11095) containing 10% fetal bovine serum, 1 mM non-essential amino acids, 10 μg/mL bovine insulin, and 1 mM sodium pyruvate at 37 °C in 5% CO_2_. Cells were passaged every three days. 

### 2.2. Inhibitor Treatment

MDA-MB-468, MCF-7, or MDA-MB-231 cells were plated at 300,000 cells per well of a six-well dish. The next day they were treated with 20 μM MG132 (Sigma C2211) for one hr, then calyculin A (Sigma C5552), okadaic acid (Alexis Biochemicals 350-011-C100), or cantharidin (Sigma C7632) for the times indicated. Cells were collected in media by scraping and centrifugation at 100 ×g for 5 min. The pellet was resuspended in 100 μL 10% trichloroacetic acid and incubated on ice for 15 min. The precipitated proteins were pelleted by centrifugation at 16,000 ×g for 15 min, washed twice with 500 μL ice cold acetone and dried at room temperature for 15 min. The pellets were resuspended in 2X SDS loading buffer, allowed to dissolve overnight, and boiled for 5 min. Protein was quantified using the BioRad DC colorimetric assay and samples were resolved on a 4-15% precast gradient gel (Biorad 345-0028). 

### 2.3. Antibodies and Immunoblotting

Proteins were transferred from gels to nitrocellulose membranes by electrophoresis for 1 h using a semi-dry transfer apparatus. Membranes were blocked with 3% bovine serum albumin (phospho antibodies) or 3% dry milk (all other antibodies) dissolved in Tris-buffered saline with 0.1% Triton X-100. Membranes were probed overnight at 4 °C with antibodies against cyclin D1 (Cell Signaling 2926), pT286 cyclin D1 (Cell Signaling 2921), actin (Sigma A2103), cyclin D3 (Cell Signaling 2936), phospho myosin light chain (Rockland 600-401-416), p70S6K (Gift from Lawrence, J.C., described in [16]). Cyclin D1, cyclin D3, and pT286 cyclin D1 and matching actin loading controls were developed using HRP-conjugated secondary antibodies (goat anti-mouse Pierce 31432 and goat anti-rabbit Pierce 31460) and SuperSignal West Pico Chemiluminescent Substrate (Thermo Fisher PI34087). Band intensities on CL-XPosure film (Thermo Fisher 34090) were below saturation and quantified using ImageJ. Other antibodies were detected using fluorescent secondary antibodies (LI-COR IRDye 800CW goat anti-mouse 926-32210 and donkey anti-rabbit 926-32213), and quantified using LI-COR Odyssey Infrared system and software. 

### 2.4. Cell Cycle Analysis

MDA-MB-468 or MCF-7 cells (1x10^6^) were treated with calyculin A for 24 hr before they were fixed with 70% ethanol overnight at 4 °C, and then washed with PBS. The cells were then incubated in 1 mL PI solution (0.1% (v/v) Triton X-100, 0.2 mg/mL DNase-free RNase A and 20 μg/mL propidium iodide in PBS) at 37 °C for 30 min. The cells were sorted on a Calibur flow cytometer for DNA content and profiles analyzed by system software. 

### 2.5. Reverse Phase Protein Array (RPPA)

RPPA analyses were done at M.D. Anderson Cancer Center. Cells were processed as described above, dissolved in 1% SDS and diluted in five 2-fold serial dilutions in dilution buffer containing 1% SDS. Serial diluted lysates were arrayed on nitrocellulose-coated slides (Grace Biolab) by Aushon 2470 Arrayer (Aushon BioSystems). Total 5,808 array spots were arranged on each slide including the spots corresponding to positive and negative controls prepared from mixed cell lysates or dilution buffer, respectively. Each slide was probed with a validated primary antibody plus a biotin-conjugated secondary antibody. Only antibodies with a Pearson correlation coefficient between RPPA and western blotting of greater than 0.7 were used in RPPA. 

The signal obtained was amplified using a Dako Cytomation-catalyzed system (Dako) and visualized by DAB colorimetric reaction. The slides were scanned, analyzed, and quantified using a customized software Microvigene (VigeneTech Inc.) to generate spot intensity. Each dilution curve was fitted with a logistic model (“Supercurve Fitting” developed by the Department of Bioinformatics and Computational Biology in M.D. Anderson Cancer Center, “http://bioinformatics.mdanderson.org/OOMPA”). This fits a single curve using all the samples (*i.e.*, dilution series) on a slide with the signal intensity as the response variable and the dilution steps as independent variable. The fitted curve is plotted with the signal intensities-both observed and fitted-on the y-axis and the log2-concentration of proteins on the x-axis for diagnostic purposes. The protein concentrations of each set of slides were then normalized by median polish, which was corrected across samples by the linear expression values using the median expression levels of all antibody experiments to calculate a loading correction factor for each sample.

## 3. Results and Discussion

### 3.1. Results

#### 3.1.1. Calyculin A Induces Depletion of Cyclin D1 in Human Breast Cancer MDA-MB-468 and MDA-MB-231 Cells

**Figure 1:**
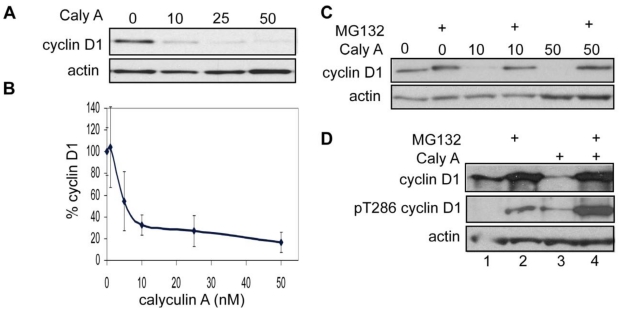
Calyculin A induces rapid proteosomal degradation of cyclin D1 human breast cancer cells. (**a**) MDA-MB-468 cells were treated with 10, 25, or 50 nM calyculin A for 30 min and cells were processed for immunoblotting with cyclin D1 and actin antibodies as described in Materials and Methods. (**b**) Data from three independent experiments are represented as mean plus or minus the standard error. (**c**) MDA-MB-468 cells were treated with 20 μM MG132 for 1 h before treatment with calyculin A for 30 min, cells were processed as described in Materials and Methods, and using 25 μg of protein per lane. (**d**) MDA-MB-231 cells were treated with DMSO alone (lane 1), 20 μM MG132 alone for 1 h (lane 2), 50 nM calyculin A alone for 30 min (lane 3), or 20 μM MG132 for 1 h follwed by 50 nM calyculin A for 30 min (lane 4). Samples of 50 μg of protein per lane were analyzed by immunoblotting for phospho T286 cyclin D1 and actin.

Because cyclin D1 is over expressed in breast cancer and phosphorylation of T286 has been implicated in control of cyclin D1 levels, we treated the triple negative (lacking ER, PR, HER2) human breast cancer cell line MDA-MB-468 with the cell permeable *Discodermia calyx* toxin calyculin A (Figure 1A and B). As seen by Western blotting >80% of endogenous cyclin D1 was depleted from cells treated with 50 nM calyculin A in 30 min compared to vehicle treated control cells (Figure 1A). Even at a dose of 10 nM calyculin A about 70% of endogenous cyclin D1 was eliminated in 30 min (Figure 1A). At concentrations lower than 10 nM, there was a dose dependent decrease in cyclin D1 levels with an EC_50_ ~3 nM (Figure 1B). A similar response to calyculin A treatment was seen with another triple negative human breast cancer cell line, MDA-MB-231 (not shown). We used the inhibitor MG132 to demonstrate that degradation of cyclin D1 induced by calyculin A was through the proteosome. Treatment of MDA-MB-468 cells with MG132 increased levels of cyclin D1 (Figure 1C lanes 1 and 2), and if added prior to calyculin A treatment (either 10 nM or 50 nM), MG132 prevented the loss of cyclin D1 (Figure 1C). Treatment of MDA-MB-231 cells with MG132 increased levels of cyclin D1 and allowed detection of phospho T286 cyclin D1 (Figure 1D lanes 1 and 2). Treatment with calyculin A alone caused near complete loss of cyclin D1, however pretreatment with MG132 preserved levels of cyclin D1 and permitted detection of a robust increase in cyclin D1 phosphorylation in response to calyculin A treatment (Figure 1D lanes 3 and 4). We observed a similar increase in phospho T286 cyclin D1 in response to combined treatment of MDA-MB-468 cells with MG132 and calyculin A (data not shown). These data show that low nanomolar concentrations of calyculin A induced the phosphorylation of T286 that promotes proteosome degradation of endogenous cyclin D1 in triple negative human breast cancer cells. We concluded that calyculin A targets the protein phosphatase that dephosphorylates T286 in cyclin D1.

#### 3.1.2. Effect of Calyculin A on Other Endogenous Phosphoproteins

**Figure 2:**
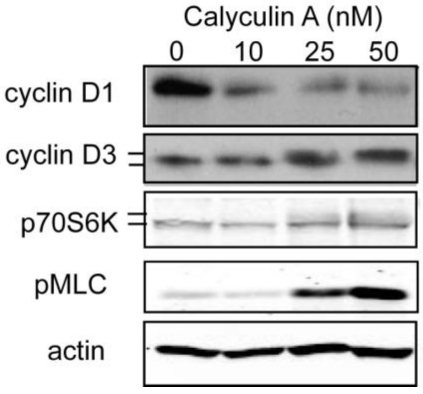
Effect of calyculin A on phosphorylation of endogenous proteins in MDA-MB-468 cells. MDA-MB-468 cells were treated with 10, 25, or 50 nM calyculin A for 30 min, processed as described in Materials and Methods and nitrocellulose membranes were blotted with antibodies against cyclin D1, total cyclin D3, total p70S6K, phospho myosin light chain, and actin. Phosphorylation of cyclin D3 and p70S6K results in slight reduction in mobility.

Calyculin A is known to inhibit both type-1 and type-2A members of the PPP phosphatase family (PP1, PP2A, PP4, PP5, PP6). We examined three different endogenous phosphoproteins in MDA-MB-468 breast cancer cells as reporters for phosphatase inhibition by low nanomolar doses of calyculin A (Figure 2). Cells were treated with vehicle alone, or 10, 25, or 50 nM calyculin A and extracts were prepared and analyzed by Western blotting. Cyclin D1 levels were substantially reduced at each concentration of calyculin A, and, once again, a dose of 10 nM was sufficient to achieve a near maximal response. Phosphorylation of cyclin D3, which reduced its electrophoretic mobility, occurred in cells treated with either 25 nM or 50 nM calyculin A, but not with 10 nM calyculin A. *In vitro* experiments showed that PP1 but not PP2A dephosphorylated cyclin D3, and PP1 associated with His-cyclin D3 in Reh, a B-cell precursor cell line [17]. Increased phosphorylation of p70S6K, as evidenced by a decrease in electrophoretic migration, was observed in cells treated with 25 or 50 nM calyculin A, but not in cells treated with 10 nM calyculin A. Dephosphorylation by p70S6K is considered to be dominantly by PP2A rather than PP1 [14,18]. Likewise, phosphorylation of myosin light chain was observed with 25 nM calyculin A and increased phosphorylation with 50 nM calyculin A, but was barely detectable and not different in cells treated with 10 nM calyculin A compared to control. Myosin light chain phosphatase is a multisubunit enzyme that has type-1 PP1c as its catalytic subunit [19]. These data show that 10 nM calyculin A was sufficient to cause a reduction in cyclin D1 levels, but not to increase phosphorylation of endogenous p70S6K, a PP2A substrate, or myosin light chain, and cyclin D3 proteins, which are PP1 substrates. These results show that calyculin A below 10 nM was highly selective for a cyclin D1 phosphatase relative to other forms of PP2A and PP1 in MDA-MB-468 cells. 

#### 3.1.3. Analysis of Calyculin A Effects on Protein Phosphorylation In Breast Cancer Cells by Reverse Phase Protein Array

**Figure 3:**
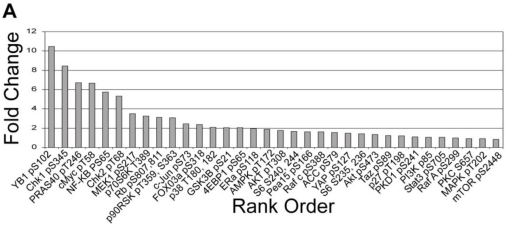
Reverse phase protein array (RPPA) analysis of effects of calyculin A on human MDA-MB-468 breast cancer cells. Cells were treated with 10 nM calyculin A for 30 min and processed as described in Materials and Methods. Average normalized staining intensity from duplicate samples of treated cells was used to calculate fold increase *vs. *vehicle treated cells in Ser/Thr phosphorylation of individual phosphosites. The fold increase in the 33 Ser/Thr phosphosites was used to generate the rank order.

Because calyculin A treatment of MDA-MB-468 cells did not affect known substrates of PP1 and PP2A, we were prompted to carry out a broader screen of changes in Ser/Thr phosphorylation. We used a reverse phase protein array, which was interrogated with 40 different phosphosite-specific antibodies (33 pSer/pThr, 7 pTyr). Duplicate samples of vehicle vs. calyculin A treated cells were analyzed and the results calculated as the -fold increase in phosphorylation and displayed as rank order, left to right (Figure 3). Calyculin A treatment of MDA-MB-468 cells only increased phosphorylation in 6 of the 40 possible sites more than a threshold set at 5-fold. No phosphorylation was decreased and phosphorylation of most of the sites only showed a small fold increase below this threshold. Phosphorylation of the protein p70S6K that was not noticeably detected by immunoblot (Figure 2) appeared in the array below this 5-fold increase. These results revealed that the calyculin A treatment increased phosphorylation of YB-1, Chk1, Chk2, cMyc, PRAS40, and NF-kB. We validated the results by immunoblotting for increased phosphorylation of PRAS40 and Chk1 in MDA-MB-468 cells treated with 10 nM calyculin A (data not shown). These results revealed highly selective inhibition of phosphatase(s) reactive with endogenous proteins by 10 nM calyculin A in human breast cancer cells.

#### 3.1.4. Effect of Okadaic Acid and Cantharidin on Cyclin D1 and Endogenous Phosphoproteins in MDA-MB-468 Breast Cancer Cells

**Figure 4:**
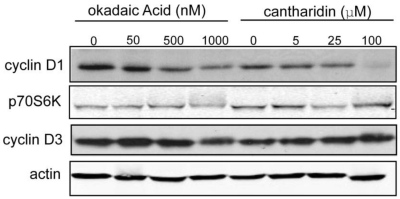
Cyclin D1 degradation in response to okadaic acid or cantharidin in MDA-MB-468 cells. MDA-MB-468 cells were treated with okadaic acid (50, 500, or 1000 nM) or cantharidin (5, 25, or 100 μM) for 1 h, and processed as described in Materials and Methods. Immunoblots were probed with antibodies against cyclin D1, p70S6K, cyclin D3, and actin.

If calyculin A caused depletion of cyclin D1 in human breast cancer cells by PPP phosphatase inhibition, one would predict that other structurally distinct toxins would elicit a similar response because active sites of type-1 and type-2A Ser/Thr PPP phosphatases are the intracellular targets of these toxins. Therefore, we examined the effects of okadaic acid and cantharidin, two other toxin phosphatase inhibitors on cyclin D1 and other phospho proteins. Cells were treated for 1 h with various doses of the toxins and the levels of cyclin D1, and electrophoretic migration of p70S6K and cyclin D3 as reporters of PP2A and PP1 activity, respectively were compared by Western blotting (Figure 4). Cyclin D1 levels were unaffected by 50 nM okadaic acid compared to controls, but as the dose was increased to 500 nM and 1000 nM there was a progressive decrease in the amount of cyclin D1. Cyclin D1 levels were reduced in cells treated with 5 μM and 25 μM cantharidin relative to controls, and there was near complete loss of cyclin D1 in cells treated with 100 μM cantharidin. These results demonstrate that three structurally different inhibitors of PPP family phosphatases produced a dose-dependent decrease in cyclin D1 levels, even though okadaic acid and cantharidin required 10 to 1000 times higher concentrations compared to calyculin A. Cyclin D1 depletion was more sensitive to these toxin inhibitors than phosphorylation of p70S6K and cyclin D3 that was only detected at the highest concentration of okadaic acid and cantharidin. This observation suggests that the cyclin D1 phosphatase has unusual sensitivity to inhibition by these toxins. 

#### 3.1.5. The Effects of Calyculin A on Cell Cycle in MDA-MB-468 Breast Cancer Cells

**Figure 5:**
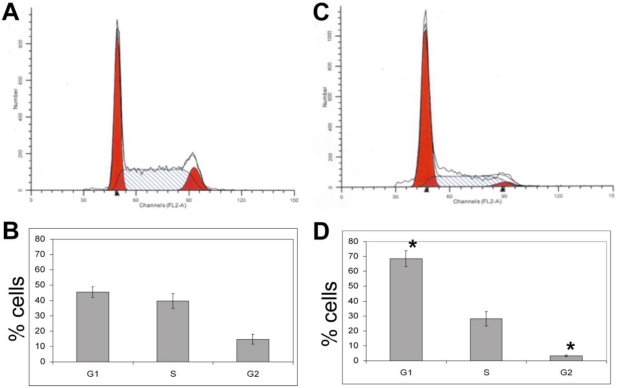
Calyculin A arrests MDA-MB-468 cells in the G_1_ phase of the cell cycle. MDA-MB-468 cells were treated with DMSO vehicle control (**a**,**b**) or 10 nM calyculin (**c**,**d**) for 24 h, fixed in 70% ethanol, stained with propidium iodide and 10,000 events were analyzed by FACS. (**a**,**c**) A gate was drawn around single cells in the dot plots where cells were sorted based on width and area. Histograms represent fraction of cells in the G_1_, S, or G_2_/M phase of the cell cycle based on DNA content. (**b**,**d**) The results from three independent experiments were averaged and represented as plus or minus the standard error for statistical significance, indicated with an asterisk.

Cell cycle status of MDA-MB-468 cells treated with calyculin A was evaluated by fluorescence activated cell sorting (FACS) analysis (Figure 5A and 5C). Cells were treated with DMSO or 10 nM calyculin A for 24 h, fixed with ethanol, and stained with the DNA dye propidium iodide prior to sorting. Some of the cells did not survive the calyculin A treatment based on staining with Trypan blue, but these cells were excluded from the gate in the FACS analysis. There was a statistically significant increase in the fraction of cells in the G_1_ (p = 0.016) phase of the cell cycle and a statistically significant decrease in the fraction of cells in the G_2_/M (p = 0.018) phase of the cell cycle in response to 10 nM calyculin A (Figure 5D *vs.* 5B). There was not a change in the fraction of cells in S phase. These results show that 10 nM calyculin A arrested MDA-MB-468 cells in the G_1_ and S phases of the cell cycle.

#### 3.1.6. Calyculin A Induces Proteasome Degradation of Cyclin D1 and Cell Cycle Arrest in MCF-7 cells

**Figure 6:**
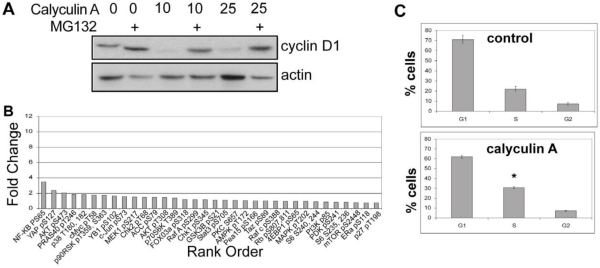
Calyculin A induces cyclin D1 proteosomal degradation and cell cycle arrest in MCF-7 cells. (**a**) MCF-7 cells were treated with vehicle or 10 or 25 nM calyculin A for 1 h with or without 1 h pretreatment with MG132 and processed for immunoblotting as described in Materials and Methods. (**b**) MCF-7 cells were treated with 10 nM calyculin A for 1 h and processed for RPPA. Average normalized staining intensity from duplicate samples of treated *vs.* control cells was used to calculate fold increase in Ser/Thr phosphorylation of individual phosphosites, used to generate the rank order. (**c**) MCF-7 cells were treated with 10 nM calyculin or DMSO vehicle control for 24 h, and 10,000 cells were analyzed by FACS for DNA content. As shown in Figure 5, the DNA content of single cells was analyzed and the results from 3 experiments averaged to achieve statistical significance represented +/- standard error.

Human breast cancer MCF-7 cells were treated with calyculin A to determine effects on cyclin D1 levels and cell cycle progression (Figure 6). Cells were pretreated with and without MG132, then vehicle alone, 10, or 25 nM calyculin A was added for 1 h, and extracts were prepared and analyzed by Western blotting. Cyclin D1 levels were extensively depleted by either concentration of calyculin A and this was prevented by pretreatment with MG132 (Figure 6A). Phosphorylation of endogenous proteins was analyzed by reverse phase protein array in response to 10 nM calyculin A for 1 h and surprisingly, none of the Ser/Thr phosphorylation sites were increased to the same extent as seen in MDA-MB-468 cells (compare Figure 6B and Figure 3). In these cells cyclin D1 was the only protein we found with a detectable change in response to the calyculin A treatment. MCF-7 cells treated with 10 nM calyculin A for 24 h showed a statistically significant (p = 0.034) increase in the fraction of cells in the S phase of the cell cycle (Figure 6C). This corresponded to a decrease in the percent of cells in the G_1_ phase (calyculin A *vs.* control) with almost no change in the G_2_/M population. These results show that 10 nM calyculin A induced proteosome degradation of cyclin D1 and cell cycle arrest in MCF-7 cells.

### 3.2. Discussion

**Figure 7:**
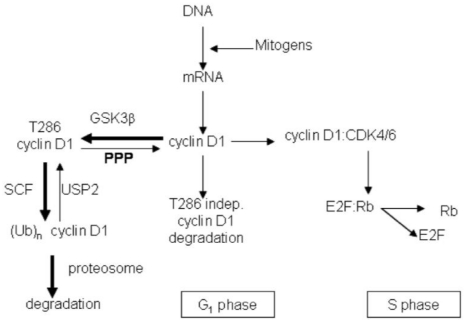
Model of Cyclin D1 action and degradation. Cyclin D1 is a subunit that binds and activates cyclin-dependent kinases CDK4/6 to phosphorylate the retinoblastoma protein Rb, to release the transcription factor E2F that is needed for cells to progress from G_1_ into S phase. Mitogens induce the accumulation of cyclin D1 via transcription in the G_1_ phase of the cell cycle. The pool of cellular cyclinD1 is controlled by a phosphorylation-dependent pathway, involving the protein Ser/Thr kinase glycogen synthase kinase 3β (GSK3β) and ubiquitination by the E3 ligase SCF. This reaction is reversible, and cyclin D1 can be deubiquitinated by USP2. Polyubiquitinated cyclin D1 is targeted for degradation by the proteosome. Cyclin D1 protein levels may also be regulated by an uncharacterized Thr286 phosphorylation-independent mechanism. Arrows are weighted to indicate rapid degradation of cyclin D1 through the phosphorylation and ubiqiutination-dependent proteosome degradation.

The results from this study demonstrate that three structurally distinct toxins, calyculin A, okadaic acid, and cantharidin, induce proteosome degradation of cyclin D1 in human breast cancer cell lines. These toxins are known to be inhibitors of PPP protein Ser/Thr phosphatases of the type-1 and type-2A classes, with some reputed preference for the type-2A enzymes (see below). Despite no similarity in structure between these toxins, the fact that they produce the same effect on cyclin D1 argues for a common Ser/Thr phosphatase as their intracellular target. The simplest explanation for our results is that the three toxins inhibit a type-2A phosphatase that dephosphorylates Thr286 in cyclin D1. An increase in cyclin D1 phosphorylation would promote SCF-mediated ubiquitination and proteasomal degradation (Figure 7). The half-life of cyclin D1 is extremely short (~30 min), a property common with a number of oncogenes such as c-myc implicating aggressive protein degradation that provides a way to regulate cellular levels of the protein [20]. In support of this hypothesis, we observed by immunoblotting an increase in phospho Thr286 cyclin D1 in MDA-MB-468 and MDA-MB-231 cells treated with calyculin A plus MG132. Inhibition of phosphatase activity without inhibition of the proteosome effectively depletes cyclin D1 in breast cancer cells and arrests them in the G_1_/S phase of the cell cycle. 

The PPP family of protein Ser/Thr phosphatases is comprised of multisubunit enzymes where catalytic subunits associate with regulatory subunits to control their activity, substrate specificity, and cellular localization [21]. The toxins used in this study are known to directly target the active sites of these enzymes. The 3-D structures of the catalytic subunit of PP1 with either okadaic acid or calyculin A and PP2A with the cyanobacterial peptide toxin microcystin-LR reveal the basis for the high affinity binding of these toxins within the Y shaped grooves that converge at the catalytic center [22,23,24]. Although the toxins bind to the phosphatase active site, they occupy different chemical space in the surface grooves and could exert substrate-specific effects in the context of phosphatase holoenzyme complexes. Assays with purified protein phosphatase catalytic subunits PP1 and PP2A showed similar concentration dependent inhibition by nanomolar calyculin A [25]. More recent biochemical studies report calyculin A inhibited PP2A with an IC_50_ of 0.25 nM, compared to PP1 and PP4 with an IC_50_ of 0.4 nM. Phosphatases PP2B, PP5 and PP7 were inhibited by calyculin A at much higher, micromolar concentrations [26]. In a study of phosphatase inhibitors added to live cells, Mumby and Bielinski (2007) concluded that the effects of calyculin A were mediated primarily through inhibition of the type-2A phosphatases [14]. Okadaic acid is well known to more potently inhibit type-2A phosphatases, relative to type-1 phosphatases that require 50 to 100 times higher concentrations [26,27,28]. Cantharidin is reported to have an IC_50_ of ~200 nM for PP2A and >1,000 nM for PP1 [26]. Taken together, these reports suggest that calyculin A, okadaic acid, and cantharidin preferentially inhibit type-2A phosphatases. Thus, our results are consistent with dephosphorylation of T286 in cyclin D1 by a type-2A phosphatase (*i.e.*, PP2A, PP4, PP6) which serves to limit phosphodegron-mediated ubiquitination and degradation by the proteosome. 

Degradation of cyclin D1 in MDA-MB-468 cells, however, was especially sensitive to calyculin A, compared to the dephosphorylation of other endogenous proteins we used as reporters of PP1 and PP2A activity. In a screen of 33 Ser/Thr phosphoproteins only 6 showed a pronounced increase in phosphorylation in response to 10 nM calyculin A. These results suggest that in the MDA-MB-468 cell line calyculin A targets a specific pool of Ser/Thr phosphatases, one of which dephosphorylates T286 in cyclin D1. One candidate is PP6 that might be inhibited by lower concentrations of calyculin A, compared to PP2A or PP1. *In vitro* phosphatase assays using purified recombinant PP6c and PP2Ac catalytic subunits showed that PP6 was more sensitive than PP2A to each of these toxins using myelin basic protein as a substrate [29]. Furthermore, previous results implicated PP6 in control of cyclin D1 levels in human prostate cancer cells [13]. On the other hand, we have used siRNA to knock down either PP6 or PP2A in MDA-MB-468 cells over 96 h, but this did not result in a decrease in cyclin D1 levels, as seen by Western blotting (data not shown). Thus, knockdown compared to calyculin A inhibition did not produce the same phenotype. Our interpretation of these results is that the remaining ~10% residual PP6 or PP2A protein in knockdown cells was sufficient to maintain cyclin D1 levels. If this is true, then 10 nM calyculin A would have to inhibit >90% of the cyclin D1 T286 phosphatase activity to trigger degradation of cyclin D1. 

The toxin dose-dependent induction of cyclin D1 degradation in MDA-MB-468 cells shows a rather surprising separation between calyculin A *vs.* okadaic acid and cantharidin. This separation contrasts dramatically from the relative *in vitro* potency of these inhibitors with type-2A phosphatases. The difference in potency for promoting cyclin D1 degradation in live cells might be attributed in part to cell permeability. The toxins are hydrophobic molecules that are cell permeable, despite having negatively charged groups. We have been unable to find comparative biophysical properties, such as logP, for these toxins in the literature. Regardless, cell permeability is unlikely to fully account for the 2 to 4 orders of magnitude difference in effectiveness of calyculin A *vs.* okadaic acid or cantharidin on cyclin D1 degradation. 

Is some other phosphoprotein affected by calyculin A involved in regulating cyclin D1 levels? Although we are not able to answer this question at this moment, it might be a direction for future studies. Maybe calyculin A at low doses increases phosphorylation of heat shock protein αB-crystallin to promote association with Fbx4 [30]. This association enhances E3 ubiquitin transferase activity for ubiquitination and degradation of cyclin D1. Alternatively, calyculin A could increase phosphorylation of S11/12 in Fbx4 to promote dimerization and activity, leading to increased degradation of cyclin D1 [31]. Another possible point of regulation in the cyclin D1 degradation pathway is the deubiquitinating enzyme USP2 (Figure 7). While phospho regulation of USP2 is not known, if phosphorylation causes USP2 inactivation, PPP inhibition would increase cyclin D1 ubiquitination and degradation. Unexpectedly, in yeast induced resistance to calyculin A *vs.* cantharidin involved different genes, indicating that binding to a common phosphatase does not fully account for cellular actions of these toxins [32]. There are derivatives of calyculin that reportedly do not inhibit PPP phosphatases [33], and it would be interesting to test whether or not these promote cyclin D1 degradation. Our results with chemically distinct toxins represent the first step toward generation of a structure-activity relationship for a specific T286 cyclin D1 phosphatase inhibitor. Such a drug based on a toxin could be valuable for treating breast cancer, in particular triple negative tumors that are refractory to current chemotherapies, as well as other types of cancer where cyclin D1 is over expressed. 

## 4. Conclusions

The toxins calyculin A, okadaic acid, and cantharidin are cell permeable inhibitors of protein Ser/Thr phosphatases of the PPP family. Calyculin A induces T286 phosphorylation and proteosome-mediated degradation of cyclin D1 in human breast cancer MDA-MB-468 and MDA-MB-231 cells. Degradation of cyclin D1 occurs at toxin concentrations below those required to alter the phosphorylation of most endogenous proteins used as reporters of phosphatase inhibition. This indicates selective inhibition of T286 cyclin D1 phosphatase that may be an effective target for pharmaceutical treatment of triple negative breast cancers. 
